# Improving response rates using a monetary incentive for patient completion of questionnaires: an observational study

**DOI:** 10.1186/1471-2288-7-12

**Published:** 2007-02-27

**Authors:** Stephen D Brealey, Christine Atwell, Stirling Bryan, Simon Coulton, Helen Cox, Ben Cross, Fiona Fylan, Andrew Garratt, Fiona J Gilbert, Maureen GC Gillan, Maggie Hendry, Kerenza Hood, Helen Houston, David King, Veronica Morton, Jo Orchard, Michael Robling, Ian T Russell, David Torgerson, Valerie Wadsworth, Clare Wilkinson

**Affiliations:** 1 Department of Health Sciences, York Trials Unit, Seebohm Rowntree Building, University of York, Heslington, York YO10 5DD, UK; 2 Department of General Practice, Cardiff University, Neuadd Meirionydd, Heath Park, Cardiff, CF14 4YS, UK; 3 Health Services Management Centre, University of Birmingham, Park House, 40 Edgbaston Park Road, Birmingham, West Midlands, B15 2TT, UK; 4 Department of Health Sciences, Seebohm Rowntree Building, University of York, Heslington, York YO10 5DD, UK; 5 Department of Psychology, Leeds Metropolitan University, Leeds LS1 3HE, UK; 6 Norwegian Knowledge Centre for Health Services, PO Box 7004, St Olavs plass, N-0130 Oslo, Norway; 7 Department of Radiology, Lillian Sutton Building, University of Aberdeen, Foresterhill, Aberdeen, AB25 2ZD, UK; 8 Cardiff University School of Medicine, North Wales Clinical School, Gwenfro Building, Unit 5, Wrexham Technology Park, Wrexham, LL13 7YP, UK; 9 South East Wales Trials Unit, Centre for Health Sciences Research, Cardiff University, Neuadd Meirionydd, Heath Park, Cardiff, CF14 4YS, UK; 10 X-ray Department, York Hospital, Wigginton Road, York, YO31 8HE, UK; 11 Institute of Medical and Social Care Research, Brigantia Building, Penrallt Road, University of Wales Bangor, Gwynedd, LL57 2AS, UK

## Abstract

**Background:**

Poor response rates to postal questionnaires can introduce bias and reduce the statistical power of a study. To improve response rates in our trial in primary care we tested the effect of introducing an unconditional direct payment of £5 for the completion of postal questionnaires.

**Methods:**

We recruited patients in general practice with knee problems from sites across the United Kingdom. An evidence-based strategy was used to follow-up patients at twelve months with postal questionnaires. This included an unconditional direct payment of £5 to patients for the completion and return of questionnaires. The first 105 patients did not receive the £5 incentive, but the subsequent 442 patients did. We used logistic regression to analyse the effect of introducing a monetary incentive to increase the response to postal questionnaires.

**Results:**

The response rate following reminders for the historical controls was 78.1% (82 of 105) compared with 88.0% (389 of 442) for those patients who received the £5 payment (diff = 9.9%, 95% CI 2.3% to 19.1%). Direct payments significantly increased the odds of response (adjusted odds ratio = 2.2, 95% CI 1.2 to 4.0, P = 0.009) with only 12 of 442 patients declining the payment. The incentive did not save costs to the trial – the extra cost per additional respondent was almost £50.

**Conclusion:**

The direct payment of £5 significantly increased the completion of postal questionnaires at negligible increase in cost for an adequately powered study.

## Background

The randomised trial is widely accepted as the most rigorous research method for minimising bias when evaluating health care technologies [1]. It is important to undertake randomised trials in primary care to produce an evidence base that is relevant to the health care delivered in that setting [2,3]. Recruitment of health care professionals and patients to randomised trials in primary care can be very difficult [4,5]. This also applies to the completion of postal questionnaires which are frequently used in trials when collecting data (including primary outcomes) from patients and practitioners [6]. Furthermore the completion of questionnaires is important for other study designs such as cohorts and surveys. These studies require informed consent but not to the same commitment as for a trial which in itself might lead to more acceptable response rates.

Poor response rates to postal questionnaires might introduce bias due to differences between responders and non-responders which limits the generalisability of results. Non-response to postal questionnaires also reduces the number of patients in a trial and therefore the power of the study to detect the hypothesised effect or precision of parameter estimates [7]. High response rates to questionnaires can enhance the quality of research by reducing bias and maximising the effective sample size, but achieving high response rates can be expensive and time-consuming [6,7].

The DAMASK Trial is a multi-centre, pragmatic randomised trial to evaluate whether patients presenting to GPs with continuing knee problems should be referred for early access to Magnetic Resonance Imaging (MRI) or directly to an orthopaedic specialist. The trial protocol has been published elsewhere [8]. Patients in our trial were asked to complete postal questionnaires at six, twelve and 24 months after randomisation. We report here our experience of identifying an effective strategy to increase response rates to postal questionnaires at twelve months.

## Methods

Postal questionnaires at twelve months from randomisation were sent to patients aged 18 to 55 with suspected internal derangement of the knee who had been recruited from general practices across North Wales, North East Scotland, and Yorkshire – areas covering urban, rural and mixed settings and a broad socio-economic spectrum. The total population of these geographical areas is around two million people registered in over six hundred general practices. For the sample size calculation we needed to follow-up 434 patients (217 direct access to MRI and 217 controls). Our previous experience of multi-centre trials in primary care suggests that 85% response rates to postal questionnaires is a realistic target so we aimed to recruit a minimum of 500 patients. Those patients who did not respond to the initial follow-up were contacted at subsequent follow-ups.

The questionnaires were twenty pages long (comprising 109 items) and asked about patients' knee-related health, general health, and health care resource use. Existing evidence about effective follow-up strategies were used to maximise response rates [6,9,10]. At six months this comprised contacting patients by post two weeks before sending the questionnaire (pre-notification), a postal reminder after two weeks, a further questionnaire after four weeks, and a telephone reminder after six weeks. All questionnaires were sent by first class post and patients were provided with a first class freepost envelope in which to return the completed questionnaire. The initial estimates of response rates at twelve months suggested that our response rates would fall short of the 85% target. Therefore, to improve response rates at twelve months we enhanced our evidence-based strategy. This was achieved by notifying patients by post two weeks before sending out the questionnaire that they would receive £5 to cover any expenses incurred for completing the questionnaire. The provision of the direct payment of £5 cash that was included with the patient questionnaire was not conditional on the questionnaire being completed. The payment was introduced after 105 patients had been approached – these patients represent historical controls. If patients did not want the £5 they returned it with the completed questionnaire. We used logistic regression using the STATA statistical package to test the effect of introducing a direct payment on the return of completed questionnaires.

To calculate the cost per response of completing a questionnaire we added the cost of questionnaires, envelopes and postage for each patient regardless of whether they responded or not to produce a total cost for each group. In addition to the total cost in the payment group there is the cost of the £5 incentive. A cost per response was achieved by dividing the total cost by the number of respondents in that group. The calculation of the marginal cost is shown in the results. Staff costs were not included as sending out questionnaires and monetary incentives were performed during normal time on the project.

Northern and Yorkshire Multi-Centre Research Ethics Committee approved this amendment to the trial protocol (reference number MREC/1/3/59).

## Results

In total, 86% (471 of 547) completed questionnaires were returned at twelve months. The mean age (SD) of respondents was 40.4 (10.1) years and 39.7% were female. Only 12 of 442 patients (3%) declined the payment of £5. Table 1 shows the proportion of patients responding for each study group. The response rate in the historical controls was 78.1% compared with 88.0% in the payment group (diff = 9.9%, 95% CI 2.3% to 19.1%). Patients in the payment group completed questionnaires quicker at the initial mailing and first reminder. The median time to respond in the historical controls was 18 days compared with 12 days in the payment group; this difference is statistically significant (P = 0.006). Table 2 shows the coefficients estimated by the logistic regression model and that direct payments to patients unconditional on the completion of questionnaires significantly increased the odds of response (odds ratio = 2.21, 95% CI 1.22 to 3.98, P = 0.009). Other factors found to be significantly associated with response were age, gender, and self-reported emotional impact of knee problem. Table 3 presents the characteristics of patients who did or did not respond and shows that younger people were less likely to complete questionnaires as well as males and people with a lower self-reported emotional impact of knee problem.

**Table 1:** Response rates by study group

**Group**	**Historical controls (n = 105)**	**Payment (n = 442)**	**Difference in response rates (CI)**
Initial mailing	39.0% (41/105)	53.8% (238/442)	14.8% (4.2 to 24.7)
1^st ^reminder	26.6% (17/64)	40.8% (84/206)	14.2% (0.6 to 25.7)
2^nd ^reminder	40.4% (19/47)	32.5% (38/117)	-7.9% (-24.2 to 7.6)
Telephone reminder	19.2% (5/26)	37.2% (29/78)	17.9% (-3.2 to 33.4)
Total	78.1% (82/105)	88.0% (389/442)	9.9% (2.3 to 19.1)

**Table 2:** Logistic regression analysis of responses (n = 547)

**Returned form**	**Coef. (B)**	**P-Value**	**Odds Ratio (95% CI)**
Payment	0.79	0.009	2.21 (1.22 to 3.98)
Age	0.05	0.000	1.05 (1.02 to 1.08)
Female	1.09	0.001	2.99 (1.57 to 5.69)
KQoL-26 physical*	0.00	0.704	1.00 (1.00 to 1.01)
KQoL-26 activities*	-0.00	0.540	1.00 (0.99 to 1.00)
KQoL-26 emotional*	0.03	0.000	1.03 (1.01 to 1.04)
Constant	-1.93	0.002	0.15 (0.04 to 0.49)

Log likelihood = -191.9; Likelihood Ratio χ^2 ^test statistic, 6 df = 52.6 (p < 0.001); Pseudo R^2 ^= 0.12

* KQoL-26 (Knee Quality of Life) is the primary outcome measure

**Table 3:** 

**Group**	**Responded**	**Age**	**KQoL-26 physical**	**KQoL-26 activities**	**KQoL-26 emotional**	**Gender**	
						
						**Male**	**Female**
Historical controls	Yes	40.8	61.5	25.8	43.4	73.7	89.7
	No	37.3	61.4	50.7	41.5	26.3	10.3
Payment	Yes	40.3	56.5	52.4	43.3	84.4	93.6
	No	33.8	53.2	38.7	29.2	15.6	6.4

The total cost for 105 patients with no incentive was £249, and the total cost for the 442 patients with a £5 incentive was £3163. The cost per response to questionnaire for the historical controls was £3.04 (£249/82) and for the direct payment group was £8.13 (£3163/389). The extra cost per trial patient of including the incentive is £4.78 (i.e. £3163/442 minus £249/105). As the gain in response from including the incentive was 0.099 (i.e. 0.880–0.781), this gives an extra cost per additional respondent of £48.28 (£4.78/0.099).

## Discussion

Edwards et al [6] showed in their meta-analyses that the odds of response to postal questionnaires were doubled when a monetary incentive (i.e. cash) was used (odds ratio 2.02; 95% CI 1.79 to 2.27) and almost doubled when incentives were not conditional on response (1.71; 1.29 to 2.26). Contacting participants before sending questionnaires also increased response (1.54; 1.24 to 1.92), as did follow-up contact (1.44; 1.22 to 1.70) and providing non-responders with a second copy of the questionnaire (1.41; 1.02 to 1.94). Our evidence-based strategy used these methods to improve response rates and was already a comprehensive strategy before introducing the monetary incentives. The inclusion of £5 direct payment incentive unconditional on response increased absolute response rates by 10%. A similar study showed that direct payment of £5 on receipt of completed questionnaire increased response rates by 12% and significantly increased odds of response (odds ratio = 1.7, 95% CI 1.1 to 2.6, p = 0.013) [11]. An evidence-based strategy that incorporates an unconditional direct payment to patients can therefore help achieve high response rates for the completion of postal questionnaires. An explanation for this has been that an unconditional incentive promotes social exchange and a sense of reciprocal obligation. In contrast, conditional incentives may change the nature of the incentive from social to economic and so easier for respondents to decline if, for example, the amount is too low [12]. The timing of using the incentive at follow-up might also be important. We implemented the incentive at the second follow-up because we met our target at the initial follow-up. Using the incentive at initial follow-up may be even more effective at improving responses and those at subsequent follow-ups. It is possible, however, that the use of direct payments in research will raise patients' expectations of this in the future. This could affect patient's altruism and reduce the effectiveness of direct payments, or require the need for larger incentives.

There is also evidence that unconditional incentives are cost-effective in non-trial settings. The advantage depends on factors such as size of the incentive, structure of study costs and the inherent interest of the questionnaire to respondents [13]. In our trial we found that the cost per completed response in the direct payment group was higher because of the addition of the £5 incentive and the extra cost per additional respondent from using the incentive was close to £50. When considering the trial budget to recruit a target of 500 patients was £650,000 at a cost of £1300 per patient (£650,000/500) an extra fifty pounds per respondent is a minimal additional cost. With regards to the size of the incentive, £5 in our study was adequate for achieving the desired effect but we could have offered a different amount to patients to cover any expenses incurred when completing questionnaires. The payment given to patients should reflect the likely time to be spent on completing the questionnaire because if the payment provided is less (or even more) than the value that the recipient places upon their time then the incentive may not have the desired effect. Maximising response rates is important for ensuring that the study has adequate power to detect a statistically significant difference between the two treatment groups. The effect of the monetary incentive on response rates in our study helped to ensure a gain in power and precision beyond what was required for the sample size calculation. The addition of an incentive, however, might affect who responds as shown in the payment group with younger people less likely to complete questionnaires as well as males and people with lower self-reported emotional impact of knee problem.

Limitations of our study include the observational design: there was no random allocation of patients to the direct payment. The incentive was introduced for patients recruited from different practices who were asked to complete questionnaires at different times in the year. This could result in a biased estimate of the effect of the incentive. We did include important prognostic variables in our regression model to control for potential confounding factors, but our findings were obtained in patients with knee problems in primary care and may not apply to other populations.

## Conclusion

The direct payment of £5 significantly increased the completion of postal questionnaires within our trial in primary care at negligible increase in cost for an adequately powered study.

## Competing interests

The author(s) declare that they have no competing interests.

## Authors' contributions

All members of the DAMASK trial team contributed to conception and design. They are SDB, CA, SB, SC, HC, BC, FF, AG, FJG, MGCG, MH, KH, HH, DK, VM, JO, MR, ITR, DT, VW and CW. SDB was responsible for writing this manuscript. All authors read, commented on, and approved the final manuscript.

## Pre-publication history

The pre-publication history for this paper can be accessed here:


